# How do professionals assess the quality of life of children with advanced cancer receiving palliative care, and what are their recommendations for improvement?

**DOI:** 10.1186/s12904-018-0328-y

**Published:** 2018-05-08

**Authors:** Josianne Avoine-Blondin, Véronique Parent, Léonor Fasse, Clémentine Lopez, Nago Humbert, Michel Duval, Serge Sultan

**Affiliations:** 1 Centre de Psycho-Oncologie, CHU Sainte-Justine, Montréal, QC H3T 1C5 Canada; 2 Department of Psychology, Université de Sherbrooke, 150, Place Charles-Le Moyne #200, Longueuil, Québec J4K 0A8 Canada; 3 Department of Psychology, Université de Bourgogne Franche-Comté, Esplanade Erasme, 21000 Dijon, France; 4 Hôpital Gustave Roussy, Villejuif, France; 5 Université Paris Descartes, Paris, France; 6 Université de Montréal, Montréal, QC Canada; 7 Department of child psychiatry, Gustave Roussy, 114, rue Édouard-Vaillant, 94805 Villejuif, France; 8 Department of Hematology/Oncology, CHU Sainte-Justine, 3175, Chemin de la Côte-Sainte-Catherine, Montréal, Québec H3T 1C5 Canada

**Keywords:** Pediatric palliative care, Quality of life, Measurement, Pediatric cancer, Qualitative study

## Abstract

**Background:**

It is known that information regarding the quality of life of a patient is central to pediatric palliative care. This information allows professionals to adapt the care and support provided to children and their families. Previous studies have documented the major areas to be investigated in order to assess the quality of life, although it is not yet known what operational criteria or piece of information should be used in the context of pediatric palliative care. The present study aims to: 1) Identify signs of quality of life and evaluation methods currently used by professionals to assess the quality of life of children with cancer receiving palliative care. 2) Collect recommendations from professionals to improve the evaluation of quality of life in this context.

**Methods:**

We selected a qualitative research design and applied an inductive thematic content analysis to the verbal material. Participants included 20 members of the Department of Hematology-Oncology at CHU Sainte-Justine from various professions (e.g. physicians, nurses, psychosocial staff) who had cared for at least one child with cancer receiving palliative care in the last year.

**Results:**

Professionals did not have access to pre-established criteria or to a defined procedure to assess the quality of life of children they followed in the context of PPC. They reported basing their assessment on the child’s non-verbal cues, relational availability and elements of his/her environment. These cues are typically collected through observation, interpretation and by asking the child, his/her parents, and other members of the care. To improve the assessment of quality of life professionals recommended optimizing interdisciplinary communication, involving the child and the family in the evaluation process, increasing training to palliative care in hematology/oncology, and developing formalized measurement tools.

**Conclusion:**

The formulation of explicit criteria to assess the quality of life in this context, along with detailed recommendations provided by professionals, support the development of systematic measurement strategy. Such a strategy would contribute to the development of common care goals and further facilitate communication between professionals and with the family.

**Electronic supplementary material:**

The online version of this article (10.1186/s12904-018-0328-y) contains supplementary material, which is available to authorized users.

## Background

Cancer is responsible for 20 to 30% of cases of children in palliative care [1–3]. Pediatric palliative care (PPC) consists of active and comprehensive care designed to prevent and alleviate suffering and improve the quality of life (QoL) of children and their families (e.g., [4–6]).

QoL is therefore at the heart of palliative care and is generally described as multidimensional and subjective [6, 7]. The physical, emotional and social aspects of QoL are the most frequently studied [8, 9]. In the specific context of PPC in oncology, recent studies of children with advanced cancer and their parents have revealed that the physical well-being of children is an inherent part of their QoL. Losses and symptoms caused by the disease on the child’s overall functioning are of great importance in this respect (e.g., [10]). In addition, more and more studies have highlighted the positive components of QoL, such as maintaining a child’s sense of normalcy and everyday pleasures (e.g., [11–14]). Importantly, spiritual dimensions have recently been studied in children and include such themes as maintaining hope and finding meaning in life (e.g., [11, 15]). In an earlier study on professionals who accompanied children in palliative care, we found unique positive dimensions to define the child’s QoL such as having fun and focusing on the present moment, feeling valued and appreciated, maintaining a sense of control and feeling that life goes on [16].

In oncology, care is focused on both children’s survival and on their overall comfort. Assessing QoL is thus particularly important as it contributes to therapeutic decisions and is useful in improving patients’ overall care (e. g., [4, 17, 18]). Several measuring tools have been developed in recent decades to assess the QoL in pediatric oncology [19–21]. However, these strategies rely heavily on coding or reporting the presence or absence of symptoms or complaints [22]. These are usually scales where the child (self-reported version) or his/her parents (proxy version) are the primary respondents [23, 24], but because these tools are focused on periods of curative treatment, they miss important topics that are specific to children with PPC.

In fact, a systematic review of the literature was recently carried out to determine whether the existing measures of QoL could be applied to this population in palliative care. The results indicate that none of the existing measures in oncology would meet the criteria for adequate use in PPC [19]. The PedsQL 4.0 for instance, which is the most widely used tool for measuring QoL in oncology, has been shown to bear shortcomings for this population as a result of inappropriate items that do not take into account the physical limitations of children in palliative care [25]. Measures of QoL do not always incorporate a temporality that is fitting for PPC, where it is recommended to focus on shorter periods of time (e.g. daily assessment) to increase the evaluator’s sensitivity to the variability of the child’s status over time [16].

In summary, the current tools do not specifically assess the QoL of children with advanced cancer who receive PPC as they do for young patients during treatment or in after-care [19, 25–27]. It has been recommended by different authors that QoL measures be developed to reflect the reality of children with a life-threatening disease [19, 26]. A tool was recently developed on the basis of areas of QoL that had been identified previously [26], but the measure is lengthy (57–65 items per version).

Considering the central importance of QoL as a target in PPC, it is noteworthy that no adequate, accurate and valid instrument is available to date. Although we may expect that professionals use their own judgments to assess the QoL, we do not know what information they use in clinical practice or how they proceed to form an opinion about the QoL of a child. One strategy to address this issue is to obtain input from professionals who have experience with these children. We need to identify how professionals evaluate the QoL of children and what are their suggestions for improving this evaluation [28].

The aim of the study is to explore and describe how professionals evaluate the QoL of the children with advanced cancer receiving PPC and what they recommend to optimize the evaluation of QoL in this area. The specific objectives are 1) to identify which signs or cues and evaluation methods are used by professionals in hematology-oncology to evaluate the QoL of children with advanced cancer receiving PPC and 2) to collect recommendations of professionals to further optimize this evaluation.

## Methods

The present study focuses on the second part of an interview taken by professionals in hematology-oncology as part of a study to define the domains of QoL in PPC [16]. The present study is based on an inductive qualitative research method within a descriptive constructivist epistemology [29, 30].

### Participants

The participants were 20 health professionals: 3 hematologist-oncologists, 1 psychiatrist, 5 nursing staff members, 2 clinical fellows, 1 nutritionist, 1 art therapist, 1 psychologist, 3 occupational therapists, and 3 physiotherapists. All were selected on the basis of the following criteria: they had to be a member of the Department of Hematology-Oncology of our hospital, have cared for at least one child (≤ 18 years) with advanced cancer receiving PPC, and speak French.

### Recruitment

The study received ethical approval from the CHU Sainte-Justine Research Ethics Committee (3547) and the University of Sherbrooke Research Ethics Committee (2013–1245). Data were collected by using maximum variation sampling recruitment strategy from professionals with diverse roles [31]. The selection of participants was based on the comprehensive list of members of the department (*N* = 103). To include different professions and avoid bias of a priori selection, a random selection was made each week to select three professionals across three different professions (physicians, nurses, other professionals). A total of 28 professionals were contacted, among whom 2 participated to a pre-test to refine interview strategies. Among the remaining 26, 23 met the inclusion criteria and 3 refused to participate (participation rate: 20/23 87% for the present analysis). The recruitment was stopped when saturation was attained across these three groups of professions. Written informed consent was collected from the participants during the initial interview. The recruitment and the interviews with participants were conducted by the first author (JAB).

### Data collection

Data collection took place from March 7, 2013 to April 2014. Individual semi-structured interviews (average duration of 1 h) were performed to collect data. The interview guide developed by the research team was inspired by Hinds et al. (2004) (see Additional file 1). The present analysis focuses on verbal material collected in response to questions specifically aimed at signs and evaluation methods used by the professionals to assess the QoL of the children in the context of PPC, and the professionals’ recommendations to optimize this assessment. Participants also completed a brief sociodemographic questionnaire. The interviews were audio recorded and transcribed for data processing.

### Data analysis

Data were analyzed inductively according to the thematic analysis approach [29, 30]. As outlined in the first report [16], data were analyzed according continual thematization process and a thematization journal were used. That way, each transcript was coded according to the two aims of the present study (aim 1: signs and evaluation approaches of QoL; aim 2: recommendations to optimize the QoL assessment). Two lists of codes were made and the process of code comparison was performed for each of these lists. The first themes were created with the aim of maintaining a low level of inference in order to respect the participants’ statements as much as possible. Thematic clusters have thus gradually taken shape and highlighted signs and evaluation methods mentioned by the professionals on the one hand (aim 1) as well as their recommendations to optimize the assessment of QoL (aim 2) on the other. A synthetic and structured representation of each of the thematic clusters was then constructed according to the two aims of the study. The analysis was carried out by hand using Word to allow greater flexibility in the analysis process. The saturation of data was attainted.

Various recommended methodological strategies were employed during the analysis [32, 33] including the systematic use of reflexive journal, triangulation, discussions and exchanges among researchers and feedback meetings with members of the hematology-oncology department. Also, by using a bottom-up process to identify the themes - which helped include comprehensive categories with higher levels of inference - we were able to ensure better validity of the results, as the emphasis from the outset was placed on the participants’ responses rather than on a simple classification.

## Results

The results highlight that professionals involved in this study do not have access to pre-established criteria or to a defined procedure that they can rely on. They were rather guided by their observations and clinical judgment.

### Aim 1. Description of signs and evaluation methods used by professionals to assess the QoL of the children with advanced cancer receiving PPC.

Thematic analysis produced three themes for signs that inform the professionals about the QoL: (1) non-verbal cues and the relational availability of the child; (2) indicators specific to domains of QoL; and (3) indicators specific to the child’s life context.

#### Non-verbal cues and the relational availability of the child

Non-verbal cues and those linked to the child’s relational availability allow professionals to form a basic idea of the child’s current overall state. Table 1 presents examples of non-verbal cues and relational availability reported by participants.

**Table 1:** Examples of non-verbal cues and relational availability to observe the quality of life

Positive	Negative
⋅ Smiles ⋅ Laughs ⋅ Better eye contact ⋅ Bright-eyed ⋅ Relaxed facial features ⋅ Relaxed body and breathing rhythm ⋅ Is awake for longer periods of time ⋅ Responds more to questions ⋅ Accepts and participates more in care ⋅ Chats more and shows a desire to interact with the environment ⋅ Is more involved in activities ⋅ Engages in his/her occupations and plays	⋅ Absent gaze ⋅ Avoids eye contact ⋅ Frowns ⋅ Body tension and restless breathing ⋅ Has difficulty calming down ⋅ Is agitated, screams, cries ⋅ Self-mutilation ⋅ Is closed off ⋅ Is curled up in bed ⋅ Appears discouraged ⋅ Responds to questions sparingly or not at all ⋅ Refuses to see professionals and to receive care ⋅ Diminished relational availability ⋅ Irritability ⋅ Sleeps most of the time

*“ […] she was suffering and you could tell by the way she was breathing and by the position of her body, in her shoulders, her arms, the fact that she was curled up, the fact that she was tense, that her face was stiff, her breathing more superficial.”* P11.

*“ I saw her smile and happy from our exchange, and she made me a little heart [on a paper], you know, she wanted to show that she was happy to feel connected. […] Otherwise, when she was not doing so well, there was no connection or empathy that could be felt or shared.”* P4.

#### Indicators specific to domains of QoL

QoL is also considered to be the result of an evaluation involving different spheres of the child’s life. Professionals refer to previously identified areas in an operationalized way: Physical comfort, Psychological alleviation, Fun and the present moment, Sense of control, Feeling that life goes on, and Meaningful social relationships [16]. For example, in “Fun and the present moment”, games are an essential criterion. Additional file 2 includes examples of signs associated with each of the dimensions of QoL.

*“ We’ll split up [the evaluation of QoL]: “Well, there is pain relief, is he okay? Yes perfect. Nutrition? Yes perfect… Does she play? Does she have fun?” In the end, it all revolves around their QoL”* P20.

#### Indicators specific to the child’s life context

Finally, most participants also mentioned taking into account signs related to the living context of the child and his/her family. These elements help professionals give meaning to other perceived signs that have been detected in the areas of QoL. The sub-themes of this theme are the individual characteristics of the child, his or her medical and care history, family dynamics, and the characteristics of his or her living environment. These aspects of the child’s environment helps contextualize and better understand the child’s QoL. Additional file 3 includes description and examples of indicators specific to the child’s life context.

Interestingly, part of the professionals’ responses focused not only on the signs themselves but on ways of accessing the signs of QoL. Subsequent to the coding work, we classified these evaluation methods into 4 themes: (1) Observation; (2) Direct investigation; (3) Interpretation; and (4) The use of diverse informants.

#### Observation

All participants referred to the observation approach, which allows professionals to identify non-verbal cues, the child’s relational availability, his or her life habits, the presence of visible symptoms or lack thereof, and contextual aspects such as the characteristics of the child’s physical environment. The observation examples reported by participants help describe this approach as a process of intentional attention directed towards the child’s and family’s discourses and daily non-verbal cues.

*“ I remember once he had celebrated his birthday and when he would talk about it, he was all smiles and, you know, he’d often be more tired and have more difficulty speaking, but when he spoke of events that had made him happy like that, it wasn’t even a big activity, really, but he had truly had fun. He would talk about it and smile […] his eyes would light up.”* P16.

#### Direct investigation

Most professionals explained that they also gather information about the child’s QoL by asking the child, parents or professionals simple and direct questions. Professionals also refer to their notes and reports on file, as well as tools to assess specific areas of QoL such as physical well-being through pain assessments.

*“[…] we would ask him: “Are you in pain?”, he was able to answer. So we could have that information […] he couldn’t elaborate on it, but…Simple questions, you know: “Are you hungry? Are you in pain?”, he could say yes or no.”* P1.

Direct investigation is an evaluation method that allows professionals to validate their first clinical impressions based on their observations and to deepen and validate their understanding of the child’s well-being and that of those around him/her.

#### Interpretation

Professionals also described the use of two types of interpretation: interpretation based on self and interpretation based on other. Interpretation in this context is a process through which professionals estimate QoL from a preliminary gathering of cues via observation or direct investigation.

#### Interpretation based on self

This sub-theme describes the appraisal of the child’s QoL based on the professional’s own points of reference and understanding. It consists of a normative judgment on the QoL of the child treated with PPC.

*“ I think that, sometimes, health professionals are misguided to speak, to qualify a good or bad quality of life because for them, it would not be a good quality of life.” P14.*

#### Interpretation based on other

In contrast, it is an approach that encourages imagining the child’s perspective, putting oneself in his or her place to understand and anticipate his or her own subjective QoL. This process comes from an intentional openness to understanding how the other may feel. Participants mentioned this form of interpretation mainly in cases where direct communication with the child was restricted.

*“ I try and imagine the child’s perspective…What brings them joy, but are no longer able to do, well I imagine that it has a big effect on their quality of life.”* (P5).

#### The use of diverse informants

The analysis of the participants’ responses highlights that most of the professionals have used the perspective of several informants to document the QoL of the child they cared for. This process allowed them to collect richer and complementary information on the child’s QoL.

*“[…] when we carry out an evaluation, we look for as much information as possible, but from different people. That means, we’ll seek out the perspective of the medical team, of the parents, and if we can ask the child, we’ll question the child.”* P1.

From this perspective, all participants mentioned the specific and individual nature of each child’s QoL and several insisted that the best informant is first and foremost the child him/herself. Great importance is also attributed to the opinion of the parents, who often provide a glimpse into the child’s inner world. Indeed, their perspective is deemed essential, especially when communication with the child is hampered by a disability or restricted because of his or her young age. Furthermore, the evaluation of QoL is reported to be more precise and accurate when the opinions of other professionals involved with the child’s care are collected.

**Fig. 1:**
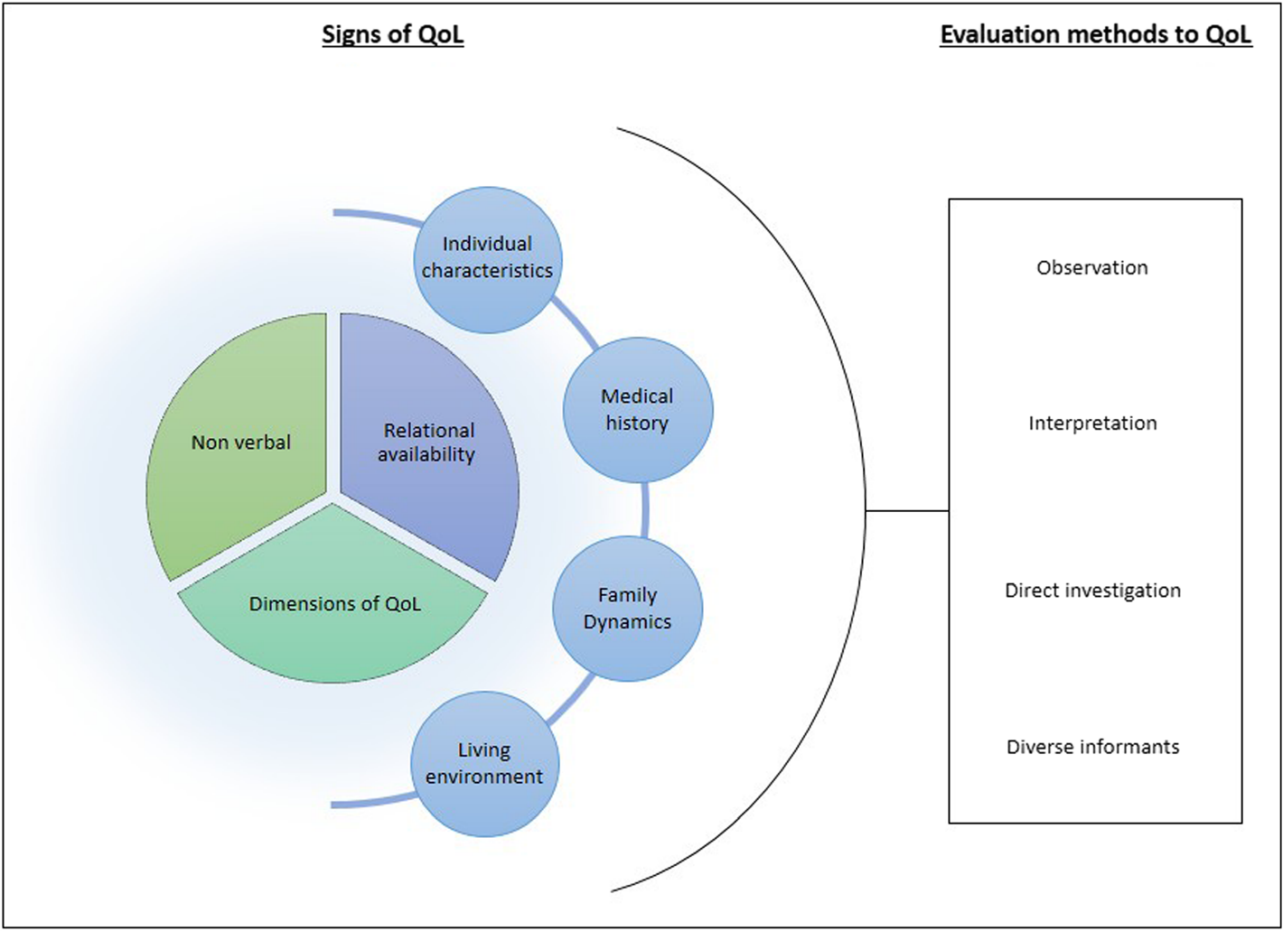
Overview of the way professionals evaluate QoL

In short, the content of the participants’ responses indicates that in clinical practice there is actually no planned and systematized evaluation of QoL. The approach is rather left to the professional’s discretion. Figure 1 illustrates an overview of the signs and ways used by professionals to assess the QoL of children they care for. This underlines that children’s QoL evaluation is complex and requires a combination of indicators, as well as a multi-source strategy to allow professionals to form an overall picture of QoL.

### Aim 2. Recommendations of professionals to further optimize the evaluation of QoL.

In this part of the interview, the health professionals mentioned several tips to improve the assessment of QoL. These tips are summarized in 4 themes: (1) promote communication among members of the health care team; (2) focus the assessment on the child’s needs, the family, and involve them in the assessment process; (3) use of a formal tool to assess the QoL; (4) develop training that is specific to PPC in oncology. Table 2 summarizes the recommendations resulting from the analysis of the content of the participants’ responses.

**Table 2:** Professionals recommendations to improve the assessment of QoL

Recommendations	Sub-themes
Promote communication among members of the health care team	⋅ Hold multidisciplinary meetings ⋅ Communicate beyond the notes on file ⋅ Collect the opinions of a meaningful professional ⋅ Involve the palliative care team
Focus the assessment on the child’s needs, the family, and involve them in the assessment process	⋅ Be attentive to the needs and desires of the family ⋅ Involve them in the assessment process
Use of a formal tool to assess the QoL	⋅ Use standardized tools in the assessment of the dimensions of QoL ⋅ Create a formal measure of QoL in PPC in oncology
Develop training that is specific to PPC in oncology	⋅ Become familiar with the context of PPC in oncology through training

#### Promote communication among members of the health care team

The professionals suggested that team meetings be held frequently and that the team put together a compilation of key information for the meaningful use of professionals. The involvement of the PPC team is also mentioned as a factor that is conducive to communication, as it helps refocus aims and establish new points of action. It was also recommended that specific moments of exchanges take place, beyond simply discussing file notes.

*“ What’s important is that everyone, even if we have our own perspective, that we manage to put it all together. So even if we do it individually and don’t look at the same aspects, in the end, it’s helpful because we will not forget the different aspects, but we have to make sure to bring back all these aspects to the team to focus on what the priority should be and ensure that no aspect has been forgotten.”* P20.

#### Focus the assessment on the child’s needs, the family, and involve them in the assessment process

The involvement of the patient and his or her family, both at the time of the assessment of QoL and in the multidisciplinary meetings, has also been described as a lever that helps create the assessment according to the families’ needs, while ensuring the validity of the assessment.

*“ […] we need to focus on the family’s needs. I may not agree with what the family or what my colleague would like for that child, but if the family says: “This is our priority.” Well, that’s what we need to focus on. […] Because if you just come in and say this is how we’re going to do things, but that it does not meet their needs, we’d be doing all that for nothing. So even if that’s not what we would have prioritized, if the family says: “This is what I want to prioritize.”, well that’s what we have to do.”* P20.

#### Use of a formal tool to assess the QoL

Professionals spontaneously mentioned the value of using an assessment tool that is specific to measuring the QoL of children with PPC, although none had ever used a formal tool or procedure in this context before.

*“ […] I think that right now, everyone is sort of using their intuition. And, we don’t really have any markers. You go with your intuition and according to the needs you see. […] we don’t have any tools…It’s really very intuitive…”* P18.

The use of such a tool would provide a common reference point to facilitate communication between patients and professionals, and among professionals of different professions in order to promote agreement and communication.

*“ I think that it’s an aspect that isn’t developed enough... it should be part of the basic stuff, just as important as a pain assessment. Relying on something that’s already out there or to come up with a new standardized test…so as to be able to quantify it and so that everyone agrees on the same system, on the same way of doing things to go in the same direction and perhaps improve his or her QoL.”* P2.

#### Develop training that is specific to PPC in oncology

The use of training that is specific to palliative care and to children with terminal cancer or who are not responding to treatment is a way for professionals to be better informed on the context, its challenges, alternative interventions, and approaches that should be favored in these difficult clinical situations. Indeed, this deeper awareness would help them better understand, evaluate, and improve the QoL of the children and their families.

*“ I think it needs to be improved perhaps by doing a little more staff training, because I really find that there isn’t a lot of training. And as for the health professionals, we are not necessarily certified. […] it is currently something that is needed, but that we do not have.”* (P9).

## Discussion

This study focuses on signs and evaluation methods of the QoL of children with advanced cancer as reported by professionals, and their recommendations to improve the assessment of QoL. Following the interviews, which we analyzed inductively using a qualitative method, we created a descriptive model that reveals the difficulty of this evaluation in current practices. Our results particularly highlight the importance of collaborative work among the multidisciplinary team members and the need for sharing and collaborating with the children and their family.

### Main findings

#### Signs and evaluation methods to assess the QoL

Based on our findings, the professionals involved in this study do not have access to pre-established criteria or to a defined procedure that they can rely on to assess the QoL of the children they follow in PPC. They are rather guided by their observations and clinical judgment.

With respect to the signs they mentioned, we can draw a parallel within the main areas of QoL (physical, psychological and social) [8, 9]. For example, professionals refer to factors such as pain levels, emotional distress, and whether the child has a supportive network. However, unlike items that are usually listed in the currently available measures of QoL, which are generally focused on deficits [22, 23], professionals reported indicators of QoL that focus on joyful moments, on anchoring oneself in the present moment, and on the pursuit of small daily accomplishments. The emphasis here is on the child’s current opportunities, in addition to his or her limitations. The spiritual component of quality of life was not explicitly flagged as an indicator by professionals in the current research. However, the professionals referred to the importance of children feeling that life goes on. A parallel can be drawn between this theme represented by indicators such as the sense of normality and achievement and those of maintaining hope and finding meaning in life which have been found to characterize the spiritual domain of QoL (e.g., [11, 15]). Importantly, to tap these domains, professionals rely on the collection of non-verbal, relational and contextual cues. This highlights the fact that information sources are varied and should be crosschecked or challenged in order to obtain a picture as complete and reliable as possible of the child’s QoL. Furthermore, the level of QoL should be adjusted according to the context, especially when the child is very sick and suffering from severe limitations. The level of QoL should in fact be considered by taking into account the individuality of the child, his or her trajectory of care and the environmental context. The need to take these contextual elements into account makes it indeed difficult to rely on a simple approach through direct assessment that only focus on the traditional dimensions of QoL (e. g., [21, 34, 35]).

The fact that no formal measure is available or used to assess the QoL in PPC leads professionals to adapt to the child’s needs according to the priorities they each perceive individually. This can lead to disagreements between different professionals regarding the QoL of a child, as the sharing of relevant information is difficult. This is reflected by the stress put by participants on communication issues. This can be problematic in an interdisciplinary work context where common goals are at the heart of the intervention. These aspects further highlight the importance of communication within teams so that individual perceptions can be shared and disparities identified and resolved [36]. As in assessing pain and emotional distress, the introduction of a formal assessment of QoL - which remains to be developed - could provide a framework for practice that promotes better communication among professionals and with the family [37].

There is a wide variety of information sources that could be used to judge the QoL according to professionals. Yet, having a variety of informants could make it difficult to identify which person should be consulted in order to evaluate the child’s QoL (the “best informant”) [9, 38, 39], although professionals insist that the child is the best suited to define his/her QoL and that a parent’s perspective is essential when communication with the child is impeded. This point of view is consistent with the current assessment procedures in pediatric oncology (e.g., [21, 34, 35]). Importantly, the analysis of the participants’ responses emphasizes the importance of their role as informants. This finding provides a new perspective on how to evaluate the QoL by showing that the diversity of their role, as well as the experience professionals acquire with the families, offer a theoretical and complementary understanding to that of the child and his/her family.

#### Professionals’ recommendations to improve the assessment of QoL

In the context of PPC, care must be coordinated among the various parties involved with the child and his/her family to enable personalized care for the child [4]. In line with this principle, communication within the multidisciplinary team is at the forefront of the improvement areas mentioned [36]. Professionals consider that communication within the health care team is essential in order to reach an agreement regarding the child’s QoL, beyond the notes that are on file. This recommendation is consistent with the acknowledged principle that interprofessional collaboration allows for the identification of shared areas across different fields, while narrowing the gap in perceptions among team members. Thus, considering that professionals from diverse professions tend to focus their approach on different areas of QoL, holding interdisciplinary meetings is a way of gaining a more complete and shared understanding of the child’s QoL. It also helps ensure the coherence of the content of their conversations when discussing care objectives with families [4, 36].

Another recommendation that professionals brought forth is to better include the child and his/her family in order to adequately reflect their needs. This recommendation is consistent with the philosophy of patient-centered care [40], where communication with the child and his/her family allows professionals to better anticipate their actions and to consider the child’s and family’s values and preferences [4, 41]. It is also a way of limiting attribution biases by professionals and ensuring that individualized assessment are carried out, beyond normative criteria alone [18].

Professionals also recommend receiving training that is specific to PPC. Indeed, it is recognized that the appropriate response to the needs of children in PPC and that of their families requires particular knowledge, skills and techniques [42, 43]. The benefits of receiving training about PPC for health care professionals has been demonstrated [44, 45]. For example, the results of a pre-test post-test study conducted with 50 pediatric clinicians who received training about PPC indicated that following training, participants reported increased confidence levels with respect to their knowledge, skills, and emotional support that they provide to children and their families [45]. It is therefore very likely that PPC-specific training allows professionals to better understand the specific reality of children with advanced cancer, thus ensuring a more accurate assessment of their QoL [44, 45].

Finally, professionals of the present study highlighted the value of creating a measurement tool for QoL that would be adapted to children receiving PPC. This would allow the assessment to be more systematized and objective, as it is currently based on the professionals’ relational skills and observations. Several advantages have previously been associated with the use of tools for measuring the QoL of children with cancer: it helps with the sharing of information among team members, improves communication with the child and the family, ensures that more needs are met, and simplifies the recording of data relevant to the child’s file [7, 18]. Recent initiatives have developed new strategies [26, 27]. While significant problems of feasibility and recruitment remain, this course of action is nevertheless promising and responds to a current need in the field. Evaluation strategies to be developed should tap the main domains identified in recent research [16].

### Strengths and limitations

The limitations of this study mainly concern the sample’s composition. 1) This study focused on the signs used by professionals to explore the QoL of children with cancer receiving PPC. The descriptive model therefore does not take into account the points of view of the children and families on QoL. However, it is informative to document professional practices. It should also be noted that the sample comprised professionals working in hematology-oncology, which excludes other clinical contexts that refer to palliative care (e.g., neonatology). 2) The distribution of our sample is also not representative of health care staff in oncology, despite the fact that we tried to include diverse occupations. Indeed, the proportion of physicians is higher than that of nurses. However, it has unlikely led to biases in the presence of certain codes or themes because the data saturation was attained across groups of professions (physicians, nurses, other professionals). 3) As in any qualitative research, self-confirmation bias cannot be ruled out. In order to prevent this problem from occurring, we intentionally used a very open collection procedure as well as methodological safeguards, including the strict upkeep of a journal and the triangulation of the research supervision.

### Implications

The present research allows us to discuss the discrepancies between current effective practices and desirable practices as mentioned by professionals. The results of this study therefore suggest the development of a personalized and more systematic evaluation of QoL. We foresee three major implications of the present findings.

First, it is undoubtedly necessary to use several information sources, including child and parents, and signs from different modalities (speech, observation, etc.) to evaluate QoL in the context of PPC, thus approaching a multi-method evaluation that is anchored in the history and current trajectory of the disease.

Second, the assessment must put the child’s feelings first and not solely rest on pre-established standards of QoL. An important notion that arises from the present findings is that of the standards or reference levels which the professionals take to compare the actual status of the child. Participants tended to, on the one hand, situate the child’s overall QoL according to signs they collect and, on the other hand, to compare this picture to the child’s previous and anticipated state with respect to his/her disease. The consequence of this observation is that the approach to assess the QoL in this context should be particularly sensitive to change, for example by focusing on a short temporal perspective such as a day, which is consistent with palliative care practices used with adults [46]. This is coherent with the recommendations of PPC standards, according to which the needs of the child and family evolve through the different stages of the disease. Thus, the assessment of needs should be a continuous, repeated process that occurs on a regular basis according to the evolution of the child’s condition [1, 4]. Feasibility and burden are core criteria for a further assessment strategy in this context.

Third, as much of the criteria used are derived from the clinical observations or judgment of professionals, they can be interpreted differently depending on the professional. This result should guide researchers towards an assessment that is validated by the child’s and family’s perceptions and by different professionals to avoid attribution bias. A proposed solution is to develop simple assessments that would allow sharing information on the central themes of QoL [16, 47].

## Conclusion

The results of this qualitative study with 20 professionals in a hematology-oncology department indicate that the assessment of QoL in PPC is currently not formalized and mainly calls for the individual judgment of professionals. Participants reported that the lack of planned or systematized procedures in regard to QoL in their care practices may lead to disagreements on the QoL of the same child in the same situation. To address these issues, professionals recommend interdisciplinary communication, involving the child and his/her family in the assessment process, developing training specific to PPC, and stress the need to create a tool to measure the QoL of children in the context of PPC specifically. Future studies should thus confirm the signs and cues to evaluate the QoL with patients and families, develop a simple and usable tool to assess the QoL. This will allow the sharing of information among professionals, child and family members on the domains relevant to the context of PPC.

## Additional files

Additional file 1: Interview Questions. Interview Questions. (PDF 15 kb)

Additional file 2: Signs associated with the dimensions of QoL from the perspective of professionals in hematology-oncology. (PDF 17 kb)

Additional file 3: Description of indicators specific to child’s life context. Description of indicators specific to child’s life context. (PDF 16 kb)

## Abbreviations

PPC: Pediatric palliative care
QoL: Quality of Life
