# Supplementary material for: How do professionals assess the quality of life of children with advanced cancer receiving palliative care, and what are their recommendations for improvement?
Source: BMC Palliat Care. 2018 May 8;17:71. doi: 10.1186/s12904-018-0328-y (PMC5938811; doi:10.1186/s12904-018-0328-y)
Supplement: Supplementary file 1 — Interview Questions. Interview Questions. (PDF 15 kb) [file 12904_2018_328_MOESM1_ESM.pdf]

Additional file 1 Interview Questions.

1. **Identifying the professional's perceptions of the child's QoL.**

Questions

According to you, what was it that made this child have a good day? A bad day? A good time? A bad time? Do you remember one of these moments?

- [Prompts]: What are the specific indications or elements that allow you to judge whether it is or was a good or bad day for the child? [a]

Now, if we think more generally, what do you think defines a good day for a child with advanced cancer in palliative care? And what defines a bad day?

- [Prompts]: What are the specific indications or elements that allow you to judge whether it is or was a good or bad day for the child? [a]

2. **Identifying the professional's perceptions of the child's QoL and activities or trends involved in defining a good or bad QoL for the child.**

Questions

Could you describe the QoL of the child for whom you provided care to?

- [Prompts]: What are the specific indications or elements that allow you to judge whether it is or was a good or bad QoL? [a]

If we think more generally about children with cancer in palliative care, what would be the criteria to consider in the evaluation of their QoL? [a]

3. **Analysis of the professionals' practices to evaluate the QoL of children with advanced cancer in PC.**

Questions

According to you, are the same criteria to assess QoL used by different professionals? Do you agree on the same child's QoL? [a]

What are your recommendations to improve this evaluation? [a]

4. **Is there anything else you would like to add concerning the QoL of children with advanced cancer in palliative care? [a]**

[a] The answers of these questions were analysed to meet the objectives of this article.
