# Supplementary material for: How do professionals assess the quality of life of children with advanced cancer receiving palliative care, and what are their recommendations for improvement?
Source: BMC Palliat Care. 2018 May 8;17:71. doi: 10.1186/s12904-018-0328-y (PMC5938811; doi:10.1186/s12904-018-0328-y)
Supplement: Supplementary file 2 — Signs associated with the dimensions of QoL from the perspective of professionals in hematology-oncology. (PDF 17 kb) [file 12904_2018_328_MOESM2_ESM.pdf]

Additional file 2. Signs associated with the dimensions of QoL from the perspective of professionals in hematology-oncology.

Dimensions of QoL	Signs	Examples from the verbatim transcripts
**Physical comfort**	· Level of pain<br>· Amount and intensity of physical symptoms and side effects of treatment (e.g., nausea, vomiting)<br>· Level of satisfaction of physiological needs (e.g., quantity and quality of sleep, ability and level of nutrition, ability to defecate, respiratory condition)	"P: […] when you're nauseous and vomit all day, well I imagine that it did not help him have a good day [...]" P3
**Psychological alleviation**	· Level of cognitive abilities (e.g., state of consciousness, cognitive impairment and associated symptoms)<br>· State of psychological health and presence of psychological symptoms related to medical condition (e.g., coping ability, level of emotional distress, presence or absence of hallucinations)	"P: Of course we weren't expecting for him to be that present or for him to be able to engage in a big conversation, but that he at least be comfortable, that he not be frightened by, by what he would see, or by his hallucinations…" P1
**Fun and the present moment**	· Ability to take advantage of the present moment and have fun<br>· Presence of shared moments of fun<br>· Ability and opportunity to engage in one or several activities the child enjoys<br>· Ability and opportunity to get pleasure out of eating	"P: Seeing him laugh, smile, um, getting pleasure out of eating, he was very very happy and he was happy to tell everyone afterwards." P7

**Sense of control**	· Ability and opportunity to control an activity in whole or in part (e.g., participate in care or lead the game)<br>· Level of physical autonomy (e.g., presence of physical disabilities, deterioration of functional abilities and limitations caused by the losses in activities of daily living)<br>· Ability and opportunity to make decisions<br>· Presence of moments of freedom and independence	"P: if you see a teenager who is bedridden, or who, you know, who is no longer autonomous, I think we all have the same thought. […] we're able to say that, you know, the patient's quality of life has probably deteriorated."P5
**Feeling that life goes on**	· Ability and opportunity to maintain activities as before the onset of the disease or as same-age peers (e.g., continue to do things like before the disease, play sports, go to the movies)<br>· Ability and opportunity to pursue achievements and accomplish things (e.g., go to school, do crafts)<br>· Ability and opportunity to achieve one or several dreams and wishes (e.g., go on a family trip, see a hockey game)	"P: […] she often talked about school, that she was going to go back to school, and you could see in her face that it was like she was seeing a bit further than just tomorrow (I: Hum hum). She was projecting herself. So, for me, it meant that she felt really good to be able to say: "Wow, I'm going to go back to school, I'm going to see my friends again.". P8
**Sense of being valued and recognized**	· The child asserts his/her needs and says he/she feels understood<br>· The child seems satisfied with the care provided<br>· The child is included in the decision-making process and informed of the follow-up<br>· An interest in the child beyond his/her symptoms is displayed	"P: […] personally to ask them […] Because I think that the best people to express what they want as a quality of life, I think, are the patients themselves and the families. […] They are best positioned to express their needs." (P2)

**Meaningful social relationships**	· Ability and opportunity to maintain meaningful relationships de (e.g., presence of the parents, siblings, friends, continuity of care)<br><br>· The child is surrounded by love and in a context of emotional security (e.g., sharing and signs of affection, the presence of care necessary to ensure safety)	"P: Is the family together? You know, if we have children who live in Chicoutimi and are hospitalized here in end-of-life care, while the rest of the family is in Chicoutimi, um that can't be easy. So, can we reunite the family if that's what they want? Actually, it's to know that family as well as possible to know their wishes. Not to believe that this is what they need, but rather understand what they really want." P20
	·	
