# Supplementary material for: How do professionals assess the quality of life of children with advanced cancer receiving palliative care, and what are their recommendations for improvement?
Source: BMC Palliat Care. 2018 May 8;17:71. doi: 10.1186/s12904-018-0328-y (PMC5938811; doi:10.1186/s12904-018-0328-y)
Supplement: Supplementary file 3 — Description of indicators specific to child’s life context. Description of indicators specific to child’s life context. (PDF 16 kb) [file 12904_2018_328_MOESM3_ESM.pdf]

Additional file 3 Description of indicators specific to child's life context.

Indicators specific to the child's life context	Description	Examples from the verbatims transcripts
**Individual characteristics of the child**	Professionals referred to the child's gender, age, lifestyle, and his or her level of satisfaction with how his/her needs and desires are being met. Knowing the child and his or her particular characteristics allows professionals to have specific benchmarks that help them better evaluate the variability of his/her QoL on a daily basis or from one encounter to the next.	"P: I remember, when she was well, she used to draw, she used to make drawings for us. She had hung up all of her little things in her room. There was a lot of stuff. So for her, I think those were the nice moments of the day (I: Ok). And you could tell that when she did that, she was happy." P8
**Medical and care history**	Factors such as the type of cancer and the trajectory of the disease. Taking these factors into account mainly provides them with benchmarks to help professionals identify the overall QoL of the child. This allows them to set reference levels or expectations on different dimensions of QoL. They look at the current progression of the child's cancer, the severity of the disease, whether there are any complications, the impact of associated treatments, lost or preserved abilities, and compare them to the child's previous condition or prognosis to determine or decide on the child's current QoL.	"P: […] This is a patient that couldn't get up, couldn't wash himself, couldn't eat. He couldn't hold a conversation, although, you know, he used to go to school the year before, you know… So, there was a gradual deterioration […] So it tells us that his QoL had deteriorated." P1
**Family dynamics**	Professionals named the impact of the social environment on the child's QoL and mentioned the influence the parents have on the child. The nature and intensity of the parents' emotional experience are thus used to interpret the level of QoL of the child.	"P: And um, the father had…he had kind of lost his…had started to sometimes become, he would scream, he would apologize afterwards, but you could tell that he was starting to suffer a lot for his daughter because we couldn't relieve her pain. And this was passed on to her a little." P8
**Living environment**	Participants mentioned concrete aspects of the child's environment as a reflection of the level of the child's QoL. For example, they referred to the fact of being at home or at the hospital in a bright, private, and colorful room, where there is as less disturbance as	"P: The bad times, often we weren't there for very long. You know, we'd read the file, we'd see that it wasn't a great day. We'd go to the room anyway to chat with mom, and, you know, often the lights were off, the curtains were

	possible. The presence of these elements is a positive sign to consider when evaluating QoL, in addition to the direct indicators already mentioned.	drawn […] That's pretty much the image I have in mind of what a bad day looked like for him." P3
